# YKL-40 is a Protective Biomarker for Fatty Liver in World Trade Center Particulate Matter-Exposed Firefighters

**DOI:** 10.4172/2155-9929.1000174

**Published:** 2014-03-21

**Authors:** Soo Jung Cho, Ghislaine C Echevarria, Young Im Lee, Sophia Kwon, Kwan Yong Park, Jun Tsukiji, William N Rom, David J Prezant, Anna Nolan, Michael D Weiden

**Affiliations:** 1 Division of Pulmonary, Critical Care and Sleep, New York University, School of Medicine, New York, USA; 2 Division of Anestesiology, School of Medicine, Pontifical Catholic University of Chile-Santiago, Chile; 3 Department of Anesthesiology, New York University School of Medicine, New York, USA; 4 Department of Environmental Medicine, New York University, School of Medicine, NY, USA; 5 Bureau of Health Services and Office of Medical Affairs, Fire Department of New York, Brooklyn, NY, USA; 6 Pulmonary Medicine Division, Department of Medicine, Montefiore Medical Center and Albert Einstein College of Medicine, Bronx, NY, USA

**Keywords:** YKL-40, Non-alcoholic fatty liver disease, Biomarker

## Abstract

**Background:**

Serum biomarkers of metabolic syndrome predict abnormal lung function in World Trade Center particulate matter (WTC-PM)-exposed Fire Department of New York (FDNY) rescue workers. In animal models, exposure to ambient PM induces non-alcoholic fatty liver disease (NAFLD), a well-known comorbidity of metabolic syndrome. YKL-40 is an inflammatory biomarker for both liver and lung disease. We tested if YKL-40 is a biomarker for NAFLD in this dust-exposed cohort.

**Methods:**

Using a nested case-control design, we studied 131 FDNY personnel who had Computer Tomography performed within 5 years post 9/11. NAFLD was defined by a liver/spleen attenuation ratio of ≤1. Serum biomarkers, lipid panel and liver function were measured in serum that had been drawn within 6 months of September 11, 2001. YKL-40 and chitotriosidase were assayed by ELISA. We tested biomarker and NAFLD association using logistic regression adjusted for age, BMI, and post-911 lung function.

**Results:**

NAFLD was present in 29/131 (22%) of the cohort. In a multivariable model increasing YKL-40 was protective while increasing triglyceride and alkaline phosphatase were risk factors for NAFLD.

**Conclusions:**

Increased YKL-40 is a protective biomarker in non-alcoholic fatty liver disease. Further studies may reveal a link between PM-induced lung and liver diseases.

## Introduction

Non-Alcoholic Fatty Liver Disease (NAFLD) refers to a spectrum of pathologic conditions involving micro- and macrovesicular steatosis in hepatocytes without other causes of chronic liver disease such as hepatitis B virus or hepatitis C virus infection and excess ethanol consumption [1]. NAFLD has emerged as the leading cause of chronic liver disease in the Western world [2,3], and affects about 30% of adults in the United States [4,5]. Its worldwide prevalence has been reported to be as high as 74% in obese individuals [6,7]. Interestingly, the prevalence of obesity/overweight in the World Trade Center (WTC) rescue worker cohort is strikingly higher than that of the general population, affecting 80% of the cohort [8,9]. We therefore investigated the prevalence of NAFLD in this cohort.

WTC-Particulate Matter (PM) exposure from the 9/11 disaster resulted in significant airflow obstruction and reactive airway disease in Fire Department of New York (FDNY) rescue workers [10-13]. We have previously reported biomarkers of metabolic syndrome that predict susceptibility to WTC PM-related lung injury [14]. Studies have also shown that ambient PM exposure to lung is closely associated with pathogenesis of metabolic syndrome [15] affecting non-pulmonary organs including the cardiovascular system [16], adipose tissue [17] and liver [18,19]. Notably, PM exposure has been shown to induce NAFLD, a hepatic manifestation of metabolic syndrome [19]. We extended our biomarker studies by identifying serum biomarkers expressed within 6 months of WTC exposure that predicted NAFLD defined on Computed Tomography (CT) scan performed years later.

The WTC-exposed FDNY cohort is a unique population whose clinical data has been systematically and rigorously captured from 1996 (pre-9/11) until the present time (post-9/11). This firefighter cohort was exposed to extremely high levels of WTC-PM (4000 times as high as the upper limit of safety range) for a brief period during rescue and recovery efforts after the WTC collapse. Firefighters with significant respiratory symptoms were referred to subspecialty pulmonary evaluation and chest CT was obtained in about 50%. Quantitative radiographic attenuation of the liver/spleen ratio seen on these CT scans defined fatty liver disease in our study [20,21].

The molecule of interest in this study is YKL-40, a chitinase-like protein of the glycosyl hydrolase family 18, [22] that is elaborated by macrophages, neutrophils, chondrocytes; fibroblasts, endothelial cells, airway epithelial cells, tumor cells and hepatic stellate cells [23]. It is interesting to note that although YKL-40 contains highly conserved chitin-binding domains, it functionally lacks chitinase activity [24], unlike chitotriosidase which is an enzymatically active chitinase produced in mature monocyte-derived macrophages, lung macrophages and Kupffer cells [25,26]. We recently reported that while increased serum chitotriosidase level is a protective biomarker of airway obstruction after WTC-PM exposure, YKL-40 was not associated with lung dysfunction post-9/11 [27]. In the current study we investigated the relationship between both YKL-40 and chitotriosidase and NAFLD in our dust-exposed firefighter population.

Although YKL-40 has been associated with disease states including hepatic fibrosis in alcoholic liver disease [28] and hepatitis C virus infection [29,30], the relationship between YKL-40 and NAFLD is unknown. We hypothesized that NAFLD in our PM-exposed cohort, as assessed by non-contrast CT scan, would be independently associated with specific serum biomarkers including YKL-40 and chitotriosidase [31]. Defining the relationship between fatty liver and YKL-40 would be a critical step in understanding the role of YKL-40 in NAFLD pathophysiology in the setting of PM exposure.

## Materials and Methods

### Nested case control design

The study cohort was derived from 1,720 exposed workers who presented to subspecialty pulmonary evaluation from October 1, 2001 and March 10, 2002. Serum was stored and available for assay (n=251); 131 of these had a non-contrast chest CT scan that routinely includes sections of the upper abdomen stopping at the adrenal glands. As a result every chest CT contained attenuation data from the liver and spleen. The chest CT was performed on average 5 years after the serum used in biomarkers assays was obtained. The protocol and informed consents were approved by the institutional review boards of Montefiore Medical Center (#07–09–320) and the human subjects review committees of New York University Medical Center Institutional Review Board (#11–00439).

### Demographics

Age, race and years of service at the FDNY were obtained from the FDNY-WTC-monitoring database. There was no evidence of alcohol abuse in the medical record of the 131 participants. Degree of exposure was self-reported at the first FDNY-WTC-monitoring and was categorized using the FDNY-WTC Exposure Intensity Index (Arrival Time): i. Presented on the morning of 9/11/2001 ii. Arrived between afternoon on 9/11/2001 and 9/12/2001 (9-11). Those arriving after day three were excluded from analysis as a result of their low numbers in this sample. Body Mass Index (BMI), blood pressure and spirometry were measured at the time of serum collection.

### Serum sampling and analysis

Blood drawn at the first post-9/11 FDNY medical monitoring exam was allowed to stand for one hour at room temperature before being centrifuged at 1,800 g for ten minutes. Serum was stored at -80°C (Bio-Reference Laboratories, Inc. Elmwood Park, NJ). Serum was thawed at 4°C and assayed using YKL-40 (MicroVue, USA), chitotriosidase (Quidel, USA) ELISA panel according to manufacturer's instructions. Lipids, glucose and liver function test were obtained from the medical records at the time of serum sampling.

### CT imaging-liver fat measurement

Each participant underwent CT scans of the chest without the administration of contrast material. Scans included images of the liver and spleen in all participants. If there were CT scans from different dates, the earliest CT images after 9/11 were examined. Primary scan data was obtained from and re-evaluated. Hepatic and splenic Hounsfield attenuation (HU) were independently measured by two experienced readers. The scan with the greatest coverage of the liver and spleen was selected for liver fat measurement: four Regions of Interest (ROI) were placed in liver and three ROI was placed in the spleen. The liver/spleen attenuation ratio (L/S ratio) was selected as the most stable measure of hepatic fat content, and was calculated by dividing the mean HU measurement of liver by the mean spleen HU measurement. The final L/S ratio was an average of the ratios obtained by the two independent readers.

### Statistical analysis

We tested normality using the Shapiro-Wilk test and Q-Q plots. We used Wilcoxon rank sum test for between group comparisons. Chi-squared test or Fisher's exact test was used for inferences on proportions.

Given the dichotomous outcome of L/S ratio we tested if biomarkers predicted NAFLD using logistic regression. Since variable YKL-40 was severely skewed, we log-transformed it before including it as continuous covariates in the model. Variables identified as potential confounders and those with a P value less than 0.2 in the univariable analysis were included in the multivariable logistic regression model. A backward stepwise approach was used to determine the most parsimonious model for the serum biomarkers, with a pre-specified P value less than 0.10. The Hosmer-Lemeshow goodness-of-fit test was used to assess calibration of the final model. The model discrimination was evaluated using the receiver operating characteristic Area under the Curve (AUC).

Data are expressed as median (interquartile range, IQR) or odds ratio (95% confidence interval), unless otherwise stated. A two-sided P-value less than 0.05 was considered significant. All analyses were performed with STATA/SE version 12.1 (StataCorp LP, College Station, TX).

## Results

### Demographics

**Figure 1:**
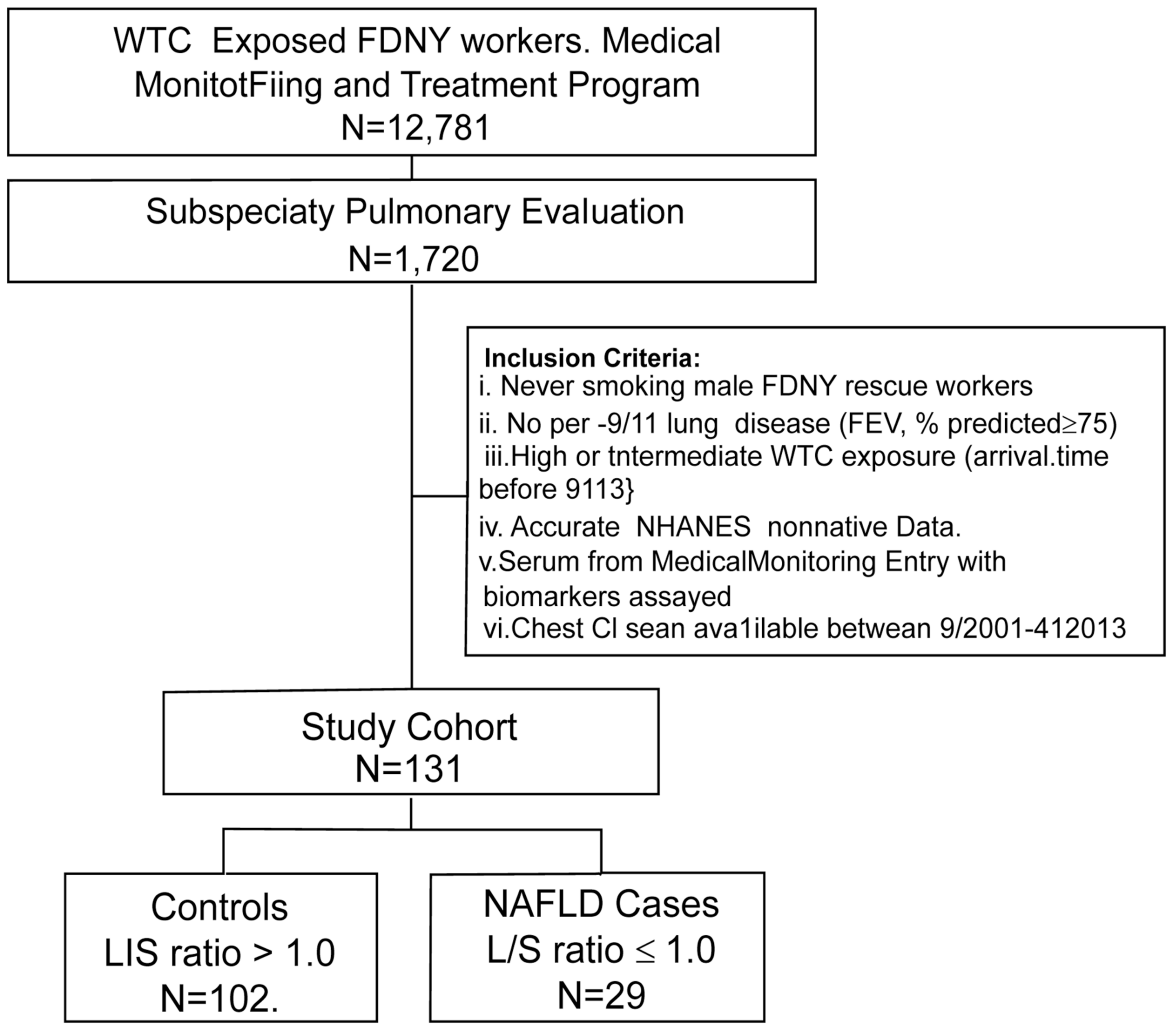
Study design. WTC: World Trade Center; FDNY: Fire Department City of New York; NHANES: National Health and Nutrition Examination Survey; L/S ratio: Liver/Spleen attenuation ratio.

Derivation of cases and controls is described in Figure 1. The prevalence of NAFLD was 22% in this study population of 131 [29 with NAFLD (L/S ratio ≤ 1) and 102 without NAFLD (L/S ratio>1)]. The median L/S ratio was 1.18 (range 0.3-1.48).

**Figure 2:**
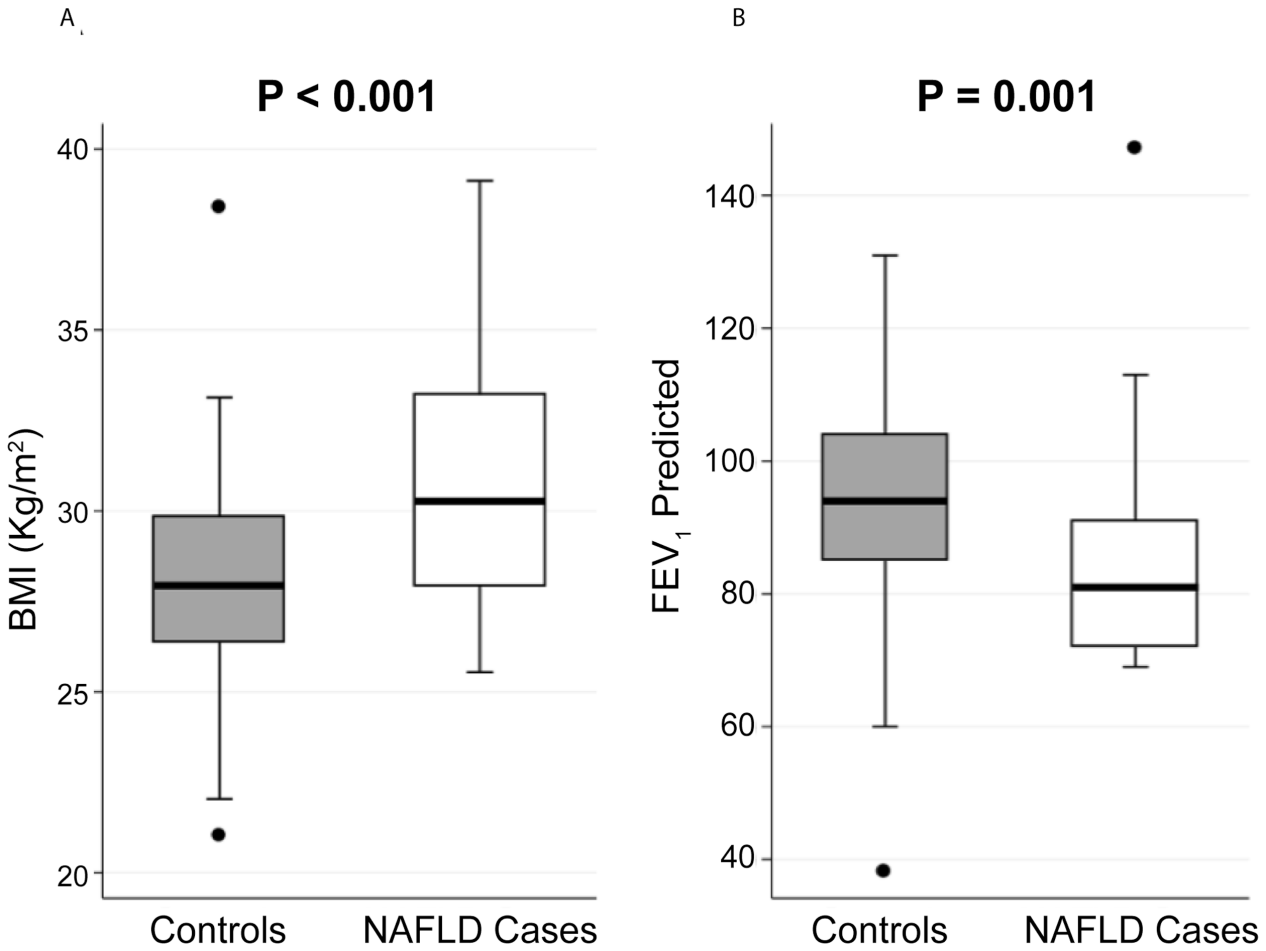
BMI (Panel A) and FEV_1_ post-911 (Panel B) are significantly different between cases (N=29) and control subjects (N=102). Results represented as box (median and interquartile range) and whiskers (10th–90th percentile). Significance determined by Wilcoxon Rank Sum test. NAFLD=Non-alcoholic fatty liver disease.

Demographics of cases and controls are shown in Table 1. All had serum biomarkers drawn after 9/11 at an initial medical monitoring exam. The median time to serum draw was 2.7 months post 9/11 and the median time to chest CT scan was 5.4 years. There were no significant differences between cases and controls in time to obtaining of serum biomarkers post-911 whereas time to chest CT scans was 6 months longer in cases (P=0.032). Cases and controls had similar age, year of service, race distribution, WTC exposure intensity, and blood pressure, and forced expiratory volume in 1 second (FEV_1_) / forced vital capacity (FVC) ratio post-/911. Cases had higher average BMI (P<0.001) and lower FEV_1_ post-9/11 than controls (P=0.001) (Figure 2).

### Serum biomarkers

Serum YKL-40, chitotriosidase, glucose, lipid panel and liver function tests were measured an average of 3 months after September 11, 2001 (Table 2). Compared to controls, cases had significantly lower YKL-40 and High-Density Lipoprotein (HDL) (P=0.028, P=0.023 respectively) and significantly higher glucose, triglyceride, Alanine Aminotransferase (ALT) Alkaline Phosphatase (ALP) and gamma-glutamyl transferase (G-GTP) levels (P=0.042, P=0.020, P=0.001 and P=0.012, respectively). There was no difference in chitotriosidase levels between cases and controls (31 ng/mL vs. 28 ng/mL, P=0.708).

### Univariable logistic model predicting L/S ratio ≤ 1

Univariable analysis was initially used to identify potential biomarkers predicting NAFLD. YKL-40, triglyceride, HDL, ALT, ALP and albumin were found to have P-values of less the 0.2 (Table 3) and were selected for multivariable analysis. There was no association between chitotriosidase and NAFLD after WTC exposure (95% CI: 0.976-1.013). Increasing YKL-40 and HDL reduced the odds of NAFLD while increasing triglyceride, ALT, ALP and albumin increased the odds of disease. In this small cohort there was no association between L/S ratio and either Low-Density Lipoprotein (LDL), Aspartate Transaminase (AST) or G-GTP.

### Multivariable logistic regression model

**Figure 3:**
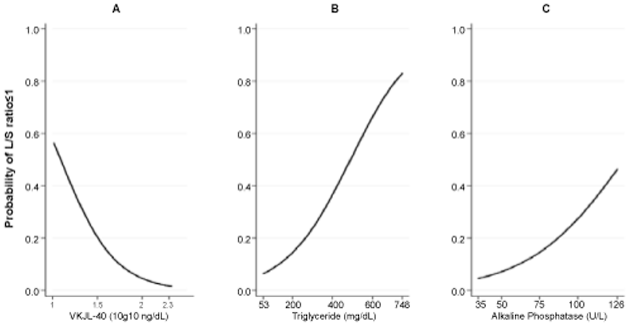
Final logistic model prediction A. Calculated probability of L/S ratio ≤ 1.0 as the concentration of YKL-40 increased over the observed biomarker range with all other covariates held constant. B. Calculated probability of L/S ratio ≤ 1.0 as triglyceride increases C. Calculated probability of L/S ratio ≤ 1.0 as ALP increases. L/S ratio: Liver/Spleen attenuation ratio; ALP: Alkaline phosphatase.

**Figure 4:**
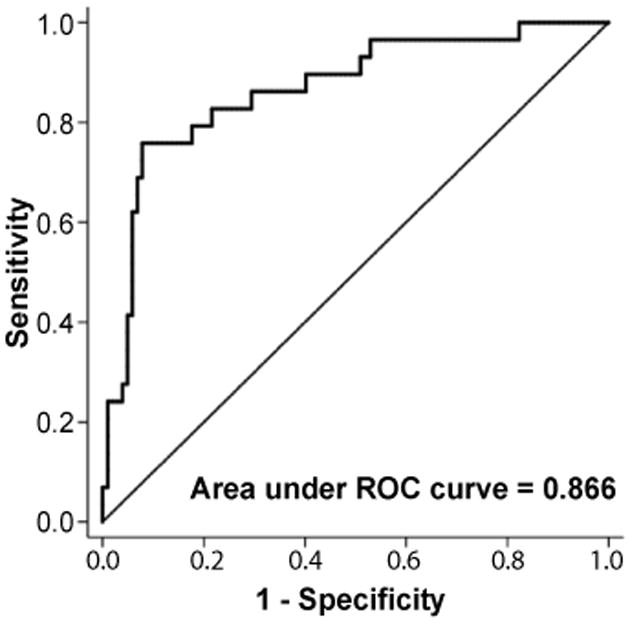
Receiver operating characteristic (ROC) of the final model to predict L/S ratio ≤ 1.0. Area under the curve (AUC) = 0.866. L/S ratio=Liver/Spleen attenuation ratio.

After adjustment for age at 9/11, BMI and FEV_1_ at the time of serum draw, the final model retained YKL-40, triglyceride and ALP as significant predictors of fatty liver (Table 3). Increasing YKL-40 reduced the odds of having NAFLD while increasing triglyceride and ALP increased the odds of having a L/S ratio ≤ 1. With every 1 log_10_ ng/mL increase in serum YKL-40, the probability of having NAFLD decreased by 42%. To determine the clinical significance of this association, we added the range of YKL-40 to the adjusted logistic model. When holding all other variables in the model constant the probability of NAFLD declined from 57% to 2% as the concentration of log_10_ YKL-40 increased from 1 to 2.33 ng/mL (Figure 3 panel A). For each 1 mg/ dL increase of triglyceride, the probability of having NAFLD decreased by 0.1%. The probability of NAFLD increased from 6% to 83% as the concentration of triglyceride increased from 53 to 748 mg/dL (Figure 3 panel B). With every 1U/L increase of ALP, the probability of having NAFLD decreased by 0.6%. The probability of NAFLD increased from 5% to 46% as the concentration of ALP increased from 35 to 126U/L (Figure 3 panel C). Area under the Receiver Operating Characteristic (ROC) of the final model to predict NAFLD was 0.866 (Figure 4).

## Discussion

The WTC collapse produced a massive acute exposure to PM causing a high incidence of respiratory dysfunction that has persisted years after 9/11 [10-13]. We have previously reported that serum biomarkers of metabolic syndrome expressed within 6 months of September 11, 2011 were associated with increased risk of abnormal FEV_1_ years later [14,32,33]. In the current study, we report that early elevation of serum YKL-40 reduces the risk of having NAFLD, the hepatic expression of metabolic syndrome, as assessed by the L/S ratio.

While 38% of firefighters in our cohort with low lung function (FEV_1_ < lower limit of normal, LLN) had NAFLD, only 12% of those with normal lung function (FEV_1_ ≥ LLN) were afflicted by fatty liver. BMI, a well-known risk factor for NAFLD, was also significantly higher in NAFLD cases. We therefore adjusted our final model for both FEV_1_ % predicted at the time of serum draw and BMI. YKL-40, triglyceride and albumin were predictive biomarkers for NAFLD after these adjustments.

It is interesting that two members of the chitinase protein family are protective biomarkers in this population. YKL-40 is a protective biomarker for NAFLD in WTC-exposed firefighters, while chitotriosidase is not. Conversely, we have previously reported that chitotriosidase, is a protective biomarker of lung dysfunction whereas YKL-40 is not [27]. Having differential associations with lung dysfunction and NAFLD may reflect distinct but overlapping pathophysiologic mechanisms of lung and liver disease after PM exposure. Such overlap is supported by our finding that elevated serum triglyceride is strongly associated with NAFLD in our cohort, just as triglyceride was found to predict abnormal FEV_1_ in our prior study [14]. Importantly, YKL-40 is enzymatically inactive and reduces risk for toxicity in an organ distant from the lung while chitotriosidase is enzymatically active and reduces risk in the organ most affected by dust inhalation. In a more general sense, ambient PM is known to induce systemic inflammation, oxidative stress response and vascular dysfunction, ultimately affecting multiple organs including liver and lungs [17,34]. The chitinase protein family may reflect counter-regulatory responses induced by PM.

Chitinases and chitinase-like proteins are active immune modulators, with chitotriosidase partaking predominantly in the innate immune system [35] while YKL-40 plays an important role in the Th2 adaptive immune response [36,37]. The effects of YKL-40 in the lung have been well described. YKL-40 over expression in a genetically modified mouse model is strongly associated with a Th2 response [38]. As Th2 cytokines are known to have an anti-inflammatory and would healing function [39], it is conceivable that YKL-40 is protective at the point of metabolic injury in the liver by curbing inflammatory processes leading to NAFLD. However, there have been no direct functional studies describing YKL-40's role in liver metabolism.

That our WTC-PM exposed patients with NAFLD had lower YLK-40 levels than controls may seem inconsistent with the observation that YKL-40 levels are usually increased in other liver diseases [28-30]. These studies were performed after liver disease was established while our investigation measured biomarker expression prior to diagnosis of NAFLD. This suggests potentially differential roles of YKL-40 in patients with a pre- or early NAFLD state versus an advanced fibrotic state of which none of our patients were diagnosed.

Our case control biomarker study has advantages over standard cross-sectional analysis in that serum biomarkers were measured on average within 3 months after acute WTC-PM exposure and CT scans were performed 5 years after serum analysis. It is therefore unlikely that our biomarkers are a consequence of the outcome. It is important to note that we have identified early biomarkers that are predictive of developing NAFLD at a later time point. However, a limitation of our study is that CT scans were not performed at the time of serum draw, precluding the assessment of disease progression or regression over time.

Our study has other limitations. Liver biopsy to pathologically confirm NAFLD was not performed due to excess risk to benefit ratio. The ratio of liver and spleen attenuation in non-contrast CT was therefore used as a noninvasive alternative to assess for fatty infiltration of the liver. We adopted the CT method for our study because it has a good correlation with histological samples [21]. Secondly, our FDNY firefighter cohort incurred massive acute exposure to WTC-PM which limits the generalizability of our findings to other study populations with less or no PM exposure. Finally, we did not have an unexposed control group to compare and therefore were unable to determine the direct effect of WTC-PM exposure on serum biomarkers. Replication of these findings in other longitudinally followed populations with and without PM exposure will ultimately be important to demonstrate the generalizability of our findings.

Increased serum YKL-40 levels were found to reduce the risk of developing NAFLD after WTC-PM exposure in a firefighter cohort, suggesting a protective role for YKL-40 in NAFLD. Further investigation is required to define if YKL-40 has a mechanistic role in mediating the protection against NAFLD after PM exposure.

## Figures and Tables

**Table 1:** Demographics.

		Controls	NAFLD Cases	P-value*
Number of Subjects		102	29	
Age at 9/11†		42 (37-46)	37 (35-45)	0.030
Years of Service at 9/11†		14 (8-19)	11 (5-16)	0.050
Race‡	Caucasian	96 (94)	29 (100)	0.215
	African American	6 (6)	0 (0)	
Intensity of WTC Exposure‡	High§	29 (28)	7 (24)	0.420
	Intermediate¶	73 (72)	22 (76)	
BMI, kg/m^2^ †		28 (26-30)	30 (28-33)	<0.001
Systolic Blood Pressure, mmHg†		114 (110-126)	118 (110-127)	0.519
Diastolic Blood Pressure, mmHg†		72 (70-80)	72 (70-80)	0.277
Time to†	Serum Collection, months	2.6 (2.1-3.2)	2.8 (2.0-3.8)	0.677
	Chest CT scan, months	61 (31-73)	68 (49-81)	0.032
Spirometry†	FEV_1_, % Predicted	94 (85-104)	81 (72-92)	0.001
	FEV_1_/FVC, %	85 (81-88)	83 (81-86)	0.067

Definition of abbreviations: BMI = body mass index; WTC = World Trade Center; FEV_1_ = forced expiratory volume in 1 second; FVC = forced vital capacity

† Values Expressed as Median (IQR).

‡ Values Expressed as N (%).

§ Arrival at WTC 9/11 Morning,

¶ Arrival at WTC between noon of 9/11 and midnight of 9/12.

* Significance assessed by Wilkoxon Rank Sum test or Chi-squared test.

**Table 2:** Serum Levels of Analytes.

Predictor	Controls† N=102	NAFLD Cases† N=29	P-value‡
YKL-40, log_10_ ng/mL	1.64 (1.5-1.8)	1.55 (1.5-1.7)	0.028
Chitotriosidase, ng/mL	31 (17-48)	28 (16-47)	0.513
Glucose, mg/dL	90 (84-95)	93 (88-100)	0.042
Triglyceride, mg/dL	135 (105-207)	226 (103-441)	0.020
HDL, mg/dL	47 (41-54)	42 (35-50)	0.027
LDL, mg/dL	131 (106-157)	130 (110-157)	0.725
ALT, U/L	30 (22-39)	39 (33-54)	0.001
AST, U/L	24 (21-28)	25 (23-32)	0.069
ALP, U/L	71 (60-82)	80 (73-85)	0.012
G-GTP, U/L	24 (19-41)	29 (21-46)	0.189
Albumin, g/L	43 (41-45)	45 (42-46)	0.168

Definition of abbreviations: OR = odds ratio; 95% CI = 95% confidence interval; HDL = high-density lipoprotein; LDL = low-density lipoprotein; ALT = alanine aminotransferase; AST = aspartate transaminase; ALP = alkaline phosphatase; G-GTP = gamma-glutamyl transpeptidase

† Values Expressed as Median (IQR).

‡ Significance assessed by Wilkoxon Rank Sum test

**Table 3:** Logistic Regression Model Predicting Liver Spleen Attenuation Ratio ≤ 1.

	Predictor	OR (95%CI)†	P-value
Univariable Analysis	YKL-40, log_10_ ng/mL	0.105 (0.016-0.694)	0.105
	Chitotriosidase, ng/mL	0.992 (0.974-1.010)	0.392
	Glucose, mg/dL	1.014 (0.991-1.037)	0.238
	Triglyceride, mg/dL	1.005 (1.002-1.008)	0.003
	HDL, mg/dL	0.963 (0.922-1.006)	0.093
	LDL, mg/dL	0.996 (0.985-1.008)	0.996
	ALT, U/L	1.016 (0.997-1.034)	0.094
	AST, U/L	1.012 (0.972-1.054)	0.548
	ALP, U/L	1.026 (1.000-1.052)	0.051
	G-GTP, U/L	1.004 (0.990-1.019)	0.553
	Albumin, g/L	1.161 (0.929-1.451)	0.188
Multivariable Analysis	YKL-40, log_10_ ng/mL	0.036 (0.003-0.427)	0.009
	Triglyceride, mg/dL	1.006 (1.002-1.011)	0.006
	ALP, U/L	1.032 (1.001-1.065)	0.046

Definition of abbreviations: OR = odds ratio; 95% CI = 95% confidence interval; HDL = high-density lipoprotein; LDL = low-density lipoprotein; ALT = alanine aminotransferase; AST = aspartate transaminase; ALP = alkaline phosphatase; G-GTP = gamma-glutamyl transpeptidase

† Adjusted for age, BMI and FEV_1_, % Predicted. Omnibus test X2 (6) = 44.349, P = <0.001. Hosmer and Lemeshow's goodness-of-fit test P=0.089. Area under ROC curve=0.866.

## Abbreviations

NAFLD: Non-alcoholic Fatty Liver Disease
FDNY: Fire Department City of New York
WTC: World Trade Center
PM: Particulate Matter
FEV_1_: Forced Expiratory Volume in 1 Second
LLN: Lower Limit of Normal
MME: Medical Monitoring Entry
SPE: Subspecialty Pulmonary Evaluation
NHANES: National Health and Nutrition Examination Survey
PFT: Pulmonary Function Test
BMI: Body Mass Index
L/S ratio: Liver/Spleen Attenuation Ratio
HDL: High-density Lipoprotein
LDL: Low-density Lipoprotein
ALT: Alanine Aminotransferase
AST: Aspartate Transaminase
ALP: Alkaline Phosphatase
G-GTP: Gamma-glutamyl Transpeptidase
